# BmP02 Atypically Delays Kv4.2 Inactivation: Implication for a Unique Interaction between Scorpion Toxin and Potassium Channel

**DOI:** 10.3390/toxins8100280

**Published:** 2016-09-27

**Authors:** Bin Wu, Yan Zhu, Jian Shi, Jie Tao, Yonghua Ji

**Affiliations:** 1 Lab of Neuropharmacology and Neurotoxicology, Shanghai University, Nanchen Road 333, Shanghai 200444, China; colabin@shu.edu.cn (B.W.); bonnie2012@shu.edu.cn (Y.Z.); 2 Vascular Biology Research Centre, Institute of Cardiovascular and Cell Sciences, St. George’s, University of London, Cranmer Terrace, London SW17 0RE, UK; 3 Central Laboratory, Putuo Hospital, Shanghai University of Traditional Chinese Medicine, 164 Lanxi road, Shanghai 200062, China

**Keywords:** scorpion toxin, voltage-gated potassium channels, electrophysiology, interaction mode

## Abstract

BmP02, a short-chain peptide with 28 residues from the venom of Chinese scorpion *Buthus martensi* Karsch, has been reported to inhibit the transient outward potassium currents (I_to_) in rat ventricular muscle cells. However, it remains unclear whether BmP02 modulates the Kv4.2 channel, one of the main contributors to I_to_. The present study investigated the effects of BmP02 on Kv4.2 kinetics and its underlying molecular mechanism. The electrophysiological recordings showed that the inactivation of Kv4.2 expressed in HEK293T cells was significantly delayed by BmP02 in a dose-response manner with EC_50_ of ~850 nM while the peak current, activation and voltage-dependent inactivation of Kv4.2 were not affected. Meanwhile, the recovery from inactivation of Kv4.2 was accelerated and the deactivation was slowed after the application of BmP02. The site-directed mutagenesis combined with computational modelling identified that K347 and K353, located in the turret motif of the Kv4.2, and E4/E5, D20/D21 in BmP02 are key residues to interact with BmP02 through electrostatic force. These findings not only reveal a novel interaction between Kv4.2 channel and its peptidyl modulator, but also provide valuable information for design of highly-selective Kv4.2 modulators.

## 1. Introduction

Ion channels, structural pores distributing in excitable as well as non-excitable cells, selectively allow ions to pass through the cell membranes. Among all, potassium channels are the largest subfamily of ion channels, mediating different potassium currents with distinct kinetic properties [1]. The transient outward potassium current (I_to_) with rapid decay, also known as the A-type potassium current (I_A_), have been observed in most neural cells and cardiac myocytes. I_to_ plays a crucial role in the repolarization of the plasma membrane following an action potential and thus indispensable for the propagation of neuronal signalling [2].

The Kv4 family of voltage-gated potassium channels includes three members (Kv4.1, Kv4.2 and Kv4.3) which rapidly activate and then immediately inactivate upon depolarization. Kv4.2 and Kv4.3 are major mediators of I_to_ or I_A_ in nervous system and heart [3,4], and their blockage or dysfunction is associated with pathologies, including pain, epilepsy, atrial fibrillation and long QT syndrome [5,6,7,8]. However, our understanding on the gating mechanism and structural characteristics of Kv4 channels is much less advanced compared to the Kv1 channel family.

Neurotoxins have proven invaluable in unravelling the structural and functional characteristics of the ion channels. One of the best-known families of them is the α-KTx isolated from scorpion venom. The toxins usually consist of 28–42 residues and contain three or four disulphide bonds [9,10]. They bind to the outer vestibule of potassium channels by interacting with hydrophobic and charged residues [11,12]. α-KTx family has already been classified into more than twenty subfamilies (α-KTx1.X to α-KTx22.X), but only one member, α-KTx15, has so far been found to target Kv4 channels [13,14].

BmP02, also known as α-KTx9.1, is a short-chain toxin purified from the venom of the Chinese scorpion (*Buthus martensi* Karsch) composed of 28 amino acids and stabilized by three disulphide bonds [15]. In spite of its structural similarity to P05, a blocker of the calcium-activated potassium channel KCa2, BmP02 barely had any effects on the KCa2 channel, suggesting that BmP02 might be quite unique in the subfamily [16].

Interestingly, BmP02 has been reported to block Kv1.3-mediate currents [17]. Our previous work showed that BmP02 could inhibit the transient outward potassium current in acute isolated rat ventricular myocytes [18], suggesting that Kv4 channels might be the potential targets of BmP02. Here, we investigated the modulatory effects and underlying molecular mechanism of BmP02 on Kv4.2.

## 2. Results

### 2.1. BmP02 Delayes the Inactivation of Kv4.2

**Figure 1:**
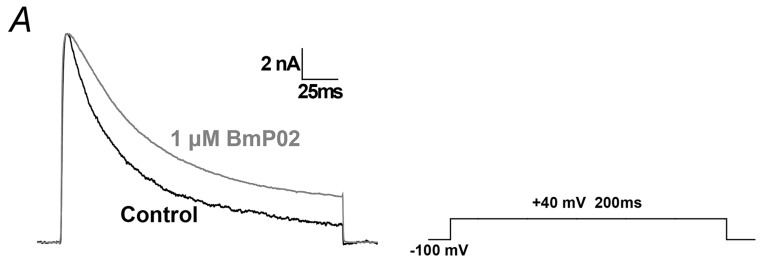

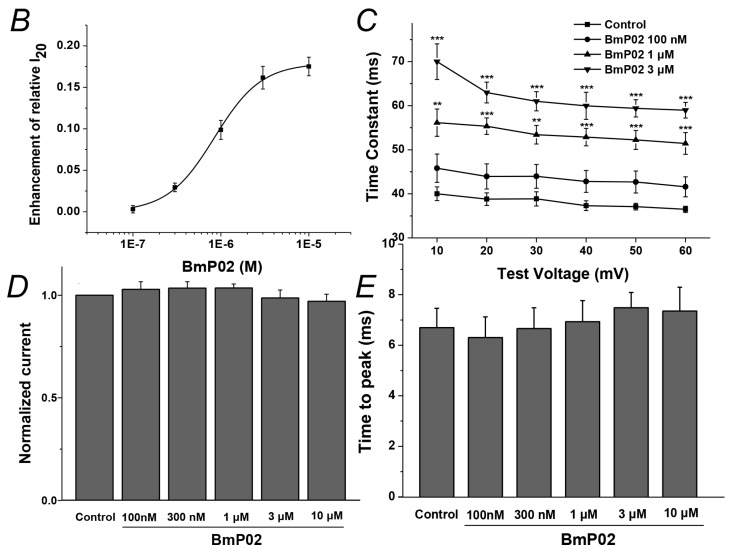
Effect of BmP02 on Kv4.2 expressed in HEK293T cells: (**A**) currents of Kv4.2 channels evoked by +40 mV pulse before (in black trace) and after the application of 3 μM BmP02 (in grey trace); (**B**) the dose–response curve of BmP02 increasing the relative I_20_ was fitted by the Hill equation. The EC_50_ value is 845.06 ± 57.32 nM. *n* = 8–10, mean ± S.E.M; (**C**) time constant of Kv4.2 inactivation evoked by different stimulus voltages from +10 mV to +60 mV before and after the application of 0.1, 1, and 3 μM BmP02, ** *p* < 0.01; *** *p* < 0.001; significant difference between control and BmP02 was tested by One-way ANOVA; (**D**) normalized peak currents evoked by +40 mV pulse before and after application of 0.1, 0.3, 1, 3, and 10 μM BmP02; and (**E**) normalized activation time evoked by +40 mV before and after application of 0.1, 0.3, 1, 3, and 10 μM BmP02.

The typical transient currents were clearly observed once the Kv4.2 channels expressed in HEK293T cells were activated by +40 mV (Figure 1A). After the application of 1 μM BmP02, the peak current and activation were not changed, but the inactivation of Kv4.2 was significantly delayed (Figure 1A). The inactivation of Kv4.2 was indicated by the relative I_20_ calculated by normalizing the current 20 ms after the onset of voltage stimulus to the peak current (relative I_20_ = I_20_/I_peak_). As shown in Figure 1B, the relative I_20_ was increased from 57.8% ± 0.5% (control, *n* = 10) to 60.7% ± 0.5% (300 nM BmP02, *n* = 10) or 75.3% ± 1.1% (10 μM BmP02, *n* = 8), respectively. The EC_50_ value for this effect was ~845 nM (Figure 1D). The time constant of inactivation was consistently increased by BmP02 (Figure 1C). In contrast, neither the amplitude of the peak current nor the activation time was affected, even in the presence of 10 μM BmP02 (Figure 1D,E).

### 2.2. BmP02 Has No Effects on the Voltage-Dependence of Kv4.2 Activation and Inactivation

**Figure 2:**
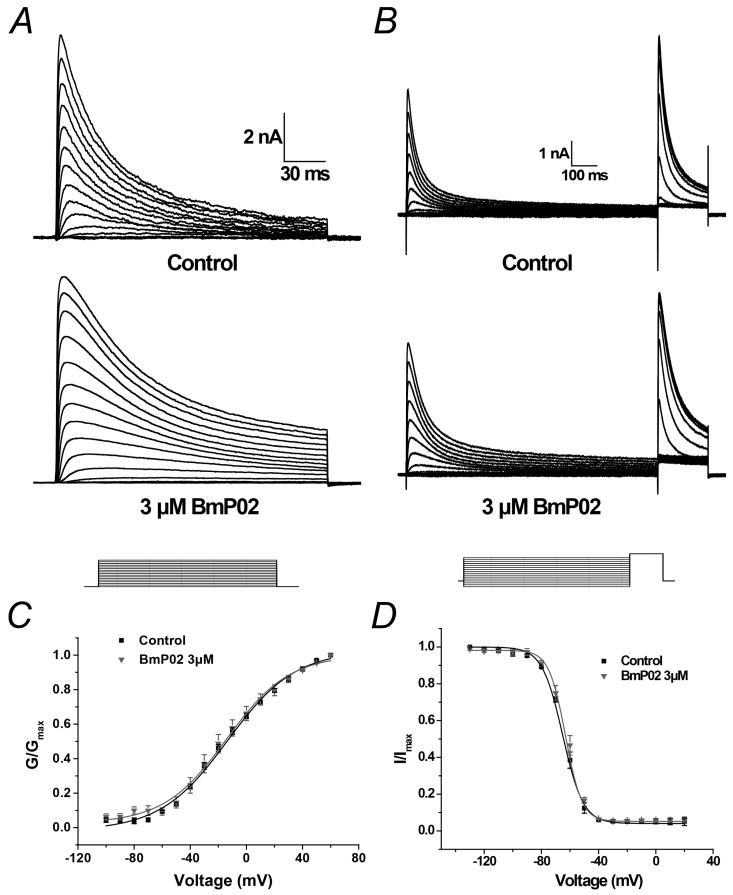
Voltage-dependent activation and inactivation of Kv4.2 before and after application of 3 μM BmP02: (**A**) representative traces of K^+^ currents elicited by stimulus voltages from −100 mV to +60 mV in 10 mV increment from holding potential of −100 mV in control (top) and in the presence of 3 μM BmP02 (bottom); (**B**) voltage dependence of steady-state inactivation determined by a two-step protocol in which a conditioning pulse to potentials ranging from −130 mV to +20 mV was followed by a test pulse to +40 mV to measure the peak current amplitudes; and (**C**,**D**) normalized G-V relationship and I-V relationship in the absence (black squares) and presence (grey triangles) of 3 μM BmP02. The fitting parameters are indicated in Table 1. *n* = 10; mean ± S.E.M.

Kv4.2 currents were elicited by the voltage from −100 to +60 mV with 10 mV increment from a holding potential of −100 mV as shown in Figure 2A. The half-maximal voltage (V_1/2_) of activation was almost not shifted by 1 μM BmP02 (from −13.87 ± 1.61 mV to −14.23 ± 1.76 mV, *n* = 10, *p* > 0.05 as shown in Figure 2C).

The steady-state inactivation was determined by a 1000 ms conditional pulse step from −140 to +20 mV prior to a test pulse at +40 mV (Figure 2B). The inactivation curve was not shifted by 1 μM BmP02 (Figure 2D). The half-maximal voltage (V_1/2_) of inactivation was −64.81 ± 1.73 mV and −62.99 ± 1.81 mV in the absence and presence of 3 μM BmP02, respectively (*n* = 10, *p* > 0.05, Figure 2D and Table 1).

### 2.3. BmP02 Accelarates the Recovery of Kv4.2 Activity from Inactivation

**Figure 3:**
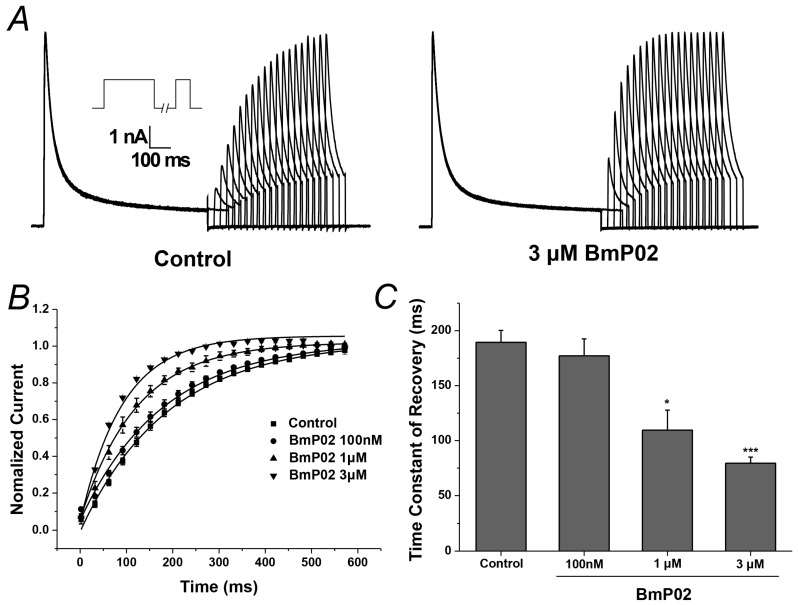
Kinetic analysis of BmP02 on the recovery of Kv4.2 activity from inactivation: *(***A***)* currents recorded at a test pulse to +40 mV for 200 ms after a variable (0–600 ms) recovery time at −120 mV following a 1000 ms conditioning prepulse at +60 mV; and *(***B**,**C***)* the recovery time course fitted to a single-exponential function to obtain the time constant of recovery. *n* = 10; mean ± S.E.M.

Kv4.2 currents determined at a test pulse to +40 mV for 200 ms after a variable (0–600 ms) recovery time at −120 mV following a 1000 ms conditioning prepulse at +40 mV were used to describe the recovery kinetics. The rate of recovery was accelerated by the application of BmP02 in a dose-dependent manner. The time constant of recovery was decreased from 189.35 ± 10.87 ms to 79.45 ± 5.62 ms by 3 μM BmP02 (*n* = 10, *p* < 0.001, Figure 3B,C and Table 2).

### 2.4. BmP02 Accelerated the Deactivation Process of Kv4.2

**Figure 4:**
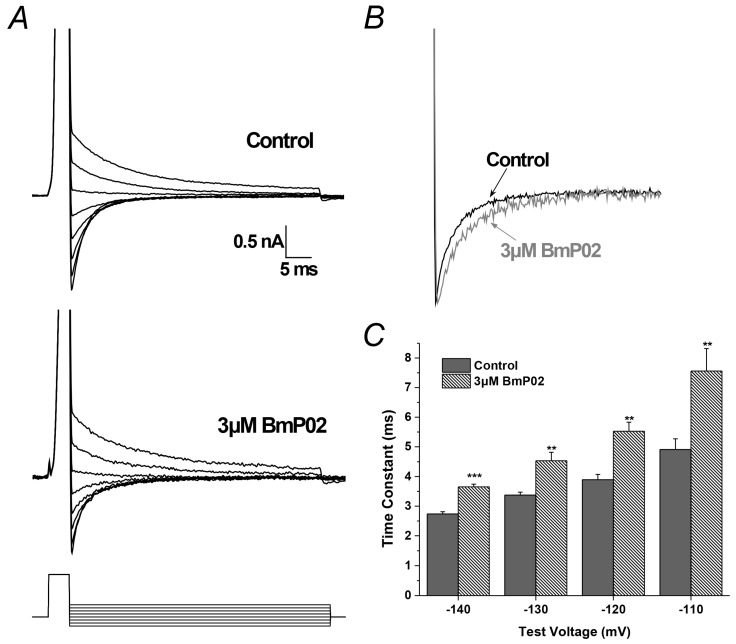
BmP02 effect on the deactivation kinectics of Kv4.2 from open state: (**A**) tail currents elicited at membrane potentials between −140 and −70 mV after a brief stimulus; (**B**) expanded view of tail currents at −140 mV in absence (black) or presence (grey) of 3 μM BmP02; and (**C**) time constants of deactivation obtained in the absence or presence of 3 μM BmP02. *n* = 8; mean ± S.E.M. ** *p* < 0.01; *** *p* < 0.001; significant difference between control and BmP02 was tested by One-way ANOVA.

Kv4.2 tail currents elicited at membrane potentials between −140 to −70 mV at the end of a 4 ms depolarization of +40 mV were used to describe the deactivation kinetics (Figure 4A). As shown in Figure 4B, the deactivation was slowed slightly by 3 μM BmP02 (Figure 4B), while the time constant of deactivation was increased after application of 3 μM BmP02 (*n* = 8, Figure 4C).

### 2.5. Amino Acid Residues Identified in Pore Region of Kv4.2 Mediate the Interaction between BmP02 and Kv4.2

**Figure 5:**
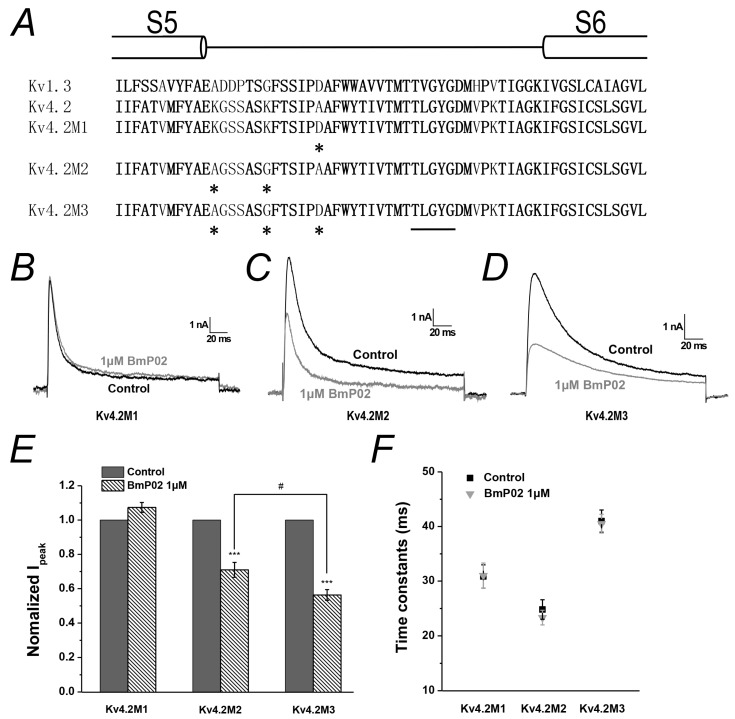
Analysis of mutants in the pore region of Kv4.2: (**A**) Sequence alignment for Kv1.3 and Kv4.2 channels. Conserved residues are shown in bold lettering. Residues (asterisks) were mutated to the corresponding residues in Kv1.3 in the three mutants. (**B**–**D**) Current traces of the three mutants, respectively, evoked by +40 mV pulses in the absence (black) or presence (grey) of 1 μM BmP02. (**E**) Normalized peak current of the three mutants evoked by +40 mV pulses in the absence (black) or presence (grey) of 1 μM BmP02. *** *p* < 0.001 significant difference between Control and BmP02, # *p* < 0.05 significant difference between Kv4.2M2 and Kv4.2M3; One-way ANOVA. (**F**) Time constants of inactivation of the three mutants in the depolarization of +40 mV before (black square) and after (grey triangle) application of 1 μM BmP02. *n* = 10; mean ± S.E.M.

Kv1.3 and Kv4.2 are considered to share a conserved structure in the selectivity filter. As reported before [19], the affinity of Kv1.3 to BmP02 was dramatically decreased when D433 in pore region was mutated into Ala. The equivalent position of D433 in Kv1.3 is A359 in Kv4.2. Except this amino acid in Kv4.2, it has also came to our attention that the distribution of charged amino acids lining the pore region of Kv1.3 or Kv4.2 is different (Figure 5A and Figure S1B) and two positively charged residues K347 and K353 only situate at the turret region of Kv4.2. Thus, the potential involvement of these three amino acid residues in mediating the interaction between BmP02 and Kv4.2 was investigated through mutagenesis. The electrophysiological recording showed that the three mutants, Kv4.2M1 (A359D), Kv4.2M2 (K347A/K353G) and Kv4.2M3 (K347A/K353G/A359D), have a similar biophysical property to the wild type Kv4.2. After the application of 1 μM BmP02, the peak current and inactivation of Kv4.2M1 were hardly changed (Figure 5B,E). However, the peak current of Kv4.2M2 or Kv4.2M3 was significantly inhibited by ~29% or ~44%, respectively (Figure 5C–E), though the inactivation of both mutants was not affected (Figure 5F).

### 2.6. The Key Residues in BmP02 for Binding to Kv4.2 Are also Determined

**Figure 6:**
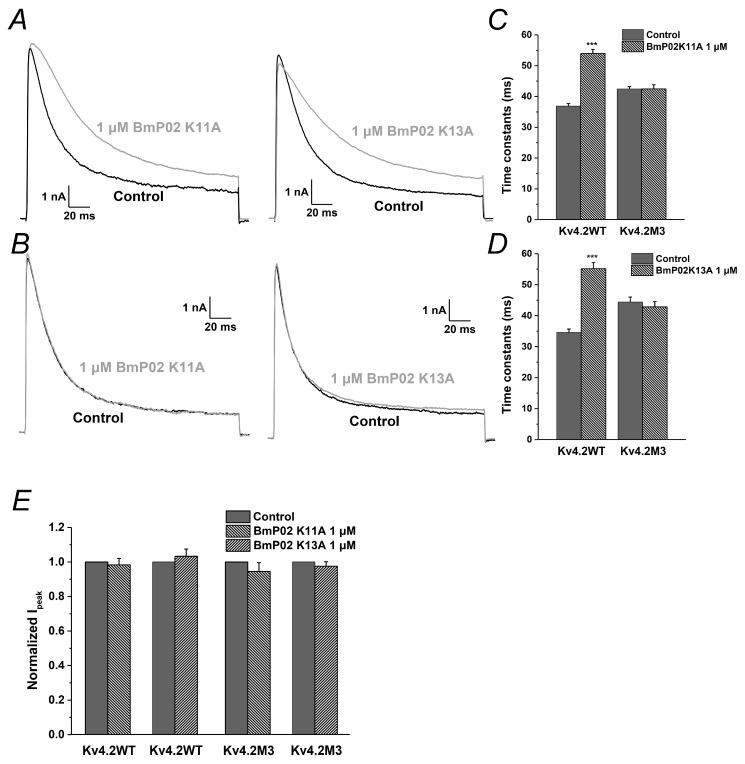
Analysis of basic amino acid residues in BmP02: (**A**) Representative currents of Kv4.2 in the absence (black) and presence (grey) of the 1 μM BmP02 K11A (left) or BmP02 K13A (right). (**B**) Representative currents of Kv4.2M3 in the absence (black) and presence (grey) of the 1 μM BmP02 K11A (left) or BmP02 K13A (right). (**C**,**D**) Time constants of inactivation of Kv4.2 and Kv4.2M3 in the depolarization of +40 mV before (black square) and after (grey triangle) application of 1 μM BmP02 K11A or BmP02 K13A, respectively. *n* = 10; mean ± S.E.M. *** *p* < 0.001, significant difference between Control and BmP02 K11A or BmP02 K13A. (**E**) Normalized peak current of the Kv4.2 or Kv4.2M3 evoked by +40 mV pulses in the absence (black) or presence (grey) of 1 μM BmP02 K11A or BmP02 K13A.

**Figure 7:**
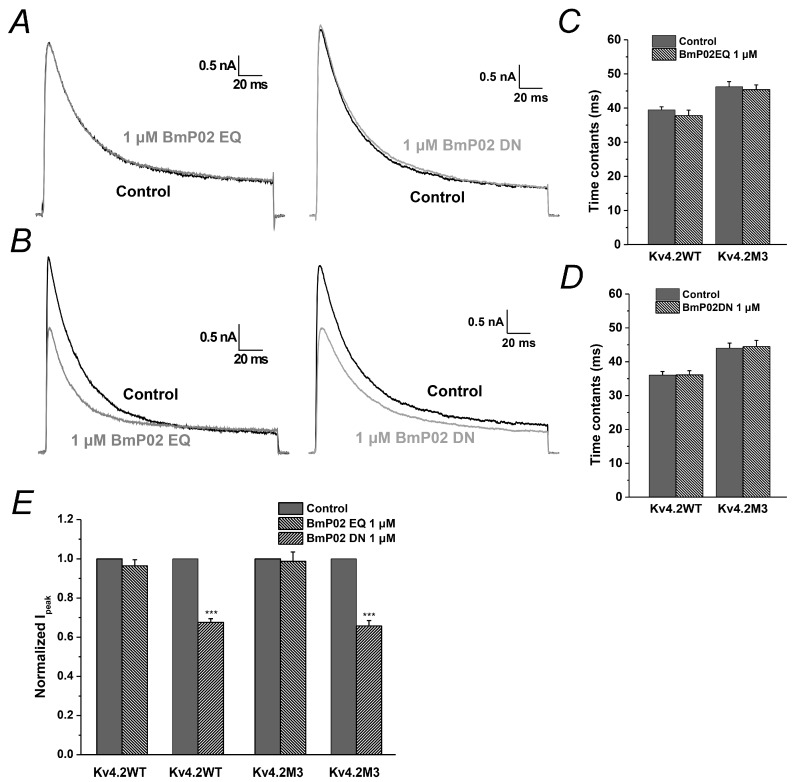
Analysis of acidic amino acid residues in BmP02: (**A**) Representative currents of Kv4.2 in the absence (black) and presence (grey) of the 1 μM BmP02EQ (left) or BmP02DN (right). (**B**) Representative currents of Kv4.2M3 in the absence (black) and presence (grey) of the 1 μM BmP02EQ (left) or BmP02 DN (right). (**C**,**D**) Time constants of inactivation of Kv4.2 and Kv4.2M3 in the depolarization of +40 mV before (black square) and after (grey triangle) application of 1 μM BmP02EQ or BmP02DN, respectively. *n* = 10; mean ± S.E.M. *** *p* < 0.001, significant difference between Control and BmP02EQ or BmP02DN. (**E**) Normalized peak current of the Kv4.2 or Kv4.2M3 evoked by +40 mV pulses in the absence (black) or presence (grey) of 1 μM BmP02EQ or BmP02DN.

As shown in the previous study [19], K11 and K13 in BmP02 are crucial for the interaction with Kv1.3. Therefore, we also explored the functional role of these two residues in targeting Kv4.2. When either K11 or K13 was mutated into alanine, the mutant failed to change the pharmacological effect of BmP02 on Kv4.2 (Figure 6A,C,D), suggesting that other residues are involved in binding to Kv4.2. In the subsequent screening of key residues, we found that E4/E5 and D20/D21 in BmP02 play pivotal roles in the interaction with Kv4.2. When E4 and E5 (BmP02EQ) were replaced by glutamine, Kv4.2 became insensitive to the peptide (Figure 7A,C). Similarly, if D20 and D21 (BmP02DN) were mutated into asparagine, the channel also lost its sensitivity to the peptide (Figure 7A,D). In contrast, these two mutants (BmP02EQ and BmP02DN) still reserve the capability of inhibiting the mutated channel Kv4.2M3 (Figure 7B,E), while the other mutants (K11A and K13A) have no pharmacological effect on Kv4.2M3 (Figure 6B,E).

### 2.7. Modelling Reveals That the Interaction between BmP02 and Kv4.2 Is Unique

**Figure 8:**
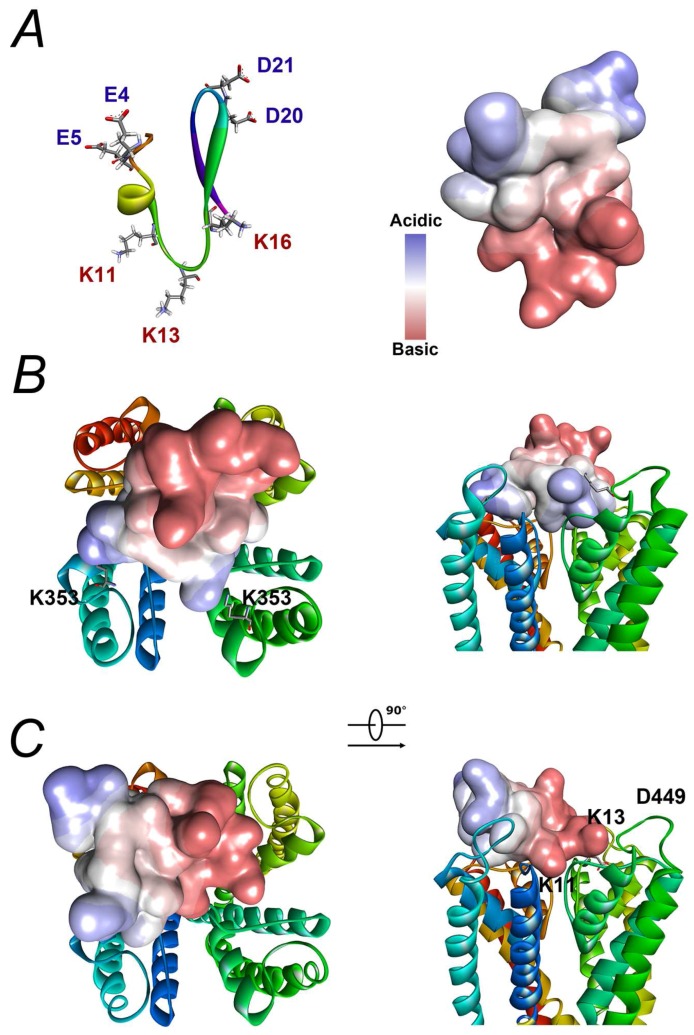
Docking analysis of BmP02 on Kv4.2 and Kv1.3: (**A**) The three-dimensional structure of BmP02 the NMR study (PDB: 1DU9). Basic acids are highlighted in red, while acidic ones are in blue (left). A molecule surface is generated to show the acid-Base Properties of BmP02 (right). (**B**) The action mode of BmP02 on Kv4.2. The highlighted K353 in two subunits determines the action mode of BmP02 described in the text. (**C**) A model of BmP02 binding to Kv1.3 is a classical model with a lysine (K11) using its side chain plugging the channel. K13 in BmP02 and D449 in Kv1.3 are highlighted.

Based on the the three-dimensional structure from the NMR study [15], BmP02 is obviously a polar molecule (Figure 8A). Two lysines (K_11_ and K_13_) situated in a large β-turn connecting the α-helix to the β-strand form one side, whereas the acidic residues located in the end of the α-helix (E_4_ and E_5_) and the turn between two β-sheets (D_20_ and D_21_) form the other side of the toxin. Docking analysis of BmP02 interaction with either Kv1.3 or Kv4.2 through computational modelling indicates that BmP02 acts like a so-called “Janus” toxin. BmP02 binds to the outer vestibules of Kv1.3 with its side chain of K_11_ plugging into the channel pore (Figure 8B and Figure S1). On the contrary, E_4_ and D_21_ are form salt bridges with the K_353_ in the turret motif of two adjacent Kv4.2 subunits, respectively (Figure 8C).

## 3. Discussion

In this study, the interaction of BmP02, a member of α-KTx9 subfamily, with Kv4.2 expressed on HEK293T cells and its molecular mechanism were investigated. In contrast to the inhibitory effect of BmP02 on Kv1.3 reported previously [17], our data showed that BmP02 delayed the inactivation of Kv4.2 in a dose-dependent manner. In other words, BmP02 seems to be a blocker to Kv1.3, but makes Kv4.2 easier to stay in the open state.

Our previous study demonstrated that BmP02 could block I_to_ in rat ventricular myocytes [18]. Considering that Kv4.2 expressed in rat heart was postulated to be a primary component of I_to_, we initially hypothesized that the current of Kv4.2 could be inhibited by BmP02. However, the surprising findings from the current study showed that he current study showed that, instead of being a blocker to Kv4.2, BmP02 significantly delayed inactivation of the channel (Figure 1), decelerated the deactivation and accelerated the recovery of Kv4.2 from inactivation. Thus, other cellular mechanisms might be involved in the inhibition of BmP02 on I_to_. A possibility is that that the channel responsible for I_to_ is partly formed by the Kv1.1 subunits co-expressed with auxiliary subunits which are sensitive to BmP02 [20,21].

The accumulating data demonstrated that the selectivity filter of the potassium channels is crucial for recognizing KTxs [12,22]. Recently, BmP02 was reported to block Kv1.3 with a high potency [17]. The capability of BmP02 blocking Kv1.3 was assumed to be from the motif consisting of three positive charged residues, H9, K11 and K13, in the toxin [19], which was also found in Kbot1 from *Buthus occitanus tunetanus* [23], and BmP03 from *Buthus martensi* Karsch [24]. If H9, K11 and K13 on BmP02 form the binding interface with Kv4.2, it is most likely that the positively charged residues (K_347_ and K_353_) located on the outer vestibule of Kv4.2 are the obstacles to binding with BmP02 due to the electrical repulsion. However, BmP02 had no effect on the inactivation of Kv4.2 when K_347_ and K_353_ in the channel were mutated. Consistently, neither K11 mutation nor K13 mutation in BmP02 can change the toxin’s effect on Kv4.2, which strongly suggests that the interaction between BmP02 and Kv4.2 might be quite unique.

Moreover, the acidic residues, such as D_433_ located on the surface of Kv1.3 vestibule are crucial for interacting with BmP02 [19]. Kv4.2 seemed to be insensitive to BmP02 when A_359_, the equivalent residue to D_433_ inn Kv1.3, was mutated into Asp, suggesting that BmP02 could use different tactics to interact with Kv1.3 or Kv4.2. The docking analysis showed that the negative charged residues E4 and D21 of BmP02, rather than positive charged H9, K11 and K13, are responsible for binding with Kv4.2. Our data from the mutagenesis also supports the possibility that the peptides will lose its effect on Kv4.2 when E4/E5 or D20/D21 are changed into Q or N, respectively. Therefore, the insensitivity of Kv4.2M1 (A_359_D) to BmP02 might attribute to the negative charged Asp repulsing BmP02.

Notably, BmP02 exerted inhibitory effect on Kv4.2 when K_347_ and K_353_ in the channel were mutated into Ala and Gly, respectively. This inhibition could be increased if A_359_ in Kv4.2 was replaced by Asp, which has also been observed in the inhibitory effect of BmP02 on Kv1.3. Thus, the distribution of positive charged residues on the outer vestibule could determine the interaction mode between BmP02 and potassium channels.

It is very intriguing to understand how BmP02 delayed the inactivation of Kv4.2 because Kv4.2 inactivates through a complicated mechanism. Our results suggested that the turret region of the channel might be a crucial factor involved in the inactivation. In addition, it has been reported that the inactivation in Kv4.2, mainly attributing to N-inactivation, occurs by a “ball-and-chain” mechanism [25]. It has been demonstrated that the pore region of ion channels would undergo conformational change after binding with KTxs [26]. It raises another possibility that the conformational change might reduce the affinity of the inactivation ball-and-chain with the pore when BmP02 binds to Kv4.2 externally. In agreement with this possibility, BmP02 accelerates the recovery of Kv4.2 from inactivation. This could attribute to the lower affinity of the inactivation ball-and-chain with the pore, which leads to a faster dissociation between them. The delay of Kv4.2 deactivation by BmP02 also suggests a coupling of channel deactivation with inactivation as previous reported [27]. Taken together, BmP02 as an unusual activator to Kv4.2 might be a novel and invaluable molecule in studying the gating mechanism and three-dimensional structure of potassium channels.

The interaction mode between BmP02 and Kv4.2 is similar to that between scorpion α-toxins and Nav channel. The major binding site of Nav channel for scorpion α-toxins is the voltage sensor in domain IV [28,29], while the key residues of Kv4.2 for interacting with BmP02 are located in the pore region. Considering that S5–S6 linker in domain I and domain IV of Nav channel is also involved in forming the typical receptor site-3 for scorpion α-toxins [30,31], the conformational change in the pore region of Nav channels is also likely to happen in the case of α-toxin binding. Therefore, it is pertinent to dissect the mechanism of the interaction between BmP02 and Kv4.2. The knowledge arising from this study might be beneficial to the general understanding about how toxins select and target ion channels, just like how Ktxs interact with potassium channels or how α-toxins interact with sodium channels.

## 4. Materials and Methods

### 4.1. Plasmids and Cell Lines

The plasmids carrying hKv4.2 (KCND2, AJ010969.1) gene and hKv1.3 (KCNC1, NM_001112741.1) genes are donated by Yanai Mei (Fudan University, Shanghai, China) and Yingliang Wu (Wuhan University, Wuhan, China), respectively. Point mutations were generated using sequential PCR with hKv4.2 channel as template. The resulting mutants are as follows: a single-residue mutant Kv4.2M1 (A359D), a double-residue mutant Kv4.2M2 (K347A/K353G) and a triple-residue mutant Kv4.2M3 (K347A/K353G/A359D). Primers were designed using Primer 5.0 (PremierBiosoft, Palo Alto, CA, USA) (Table S1).

All experiments were performed on HEK293T cell lines. HEK293T cells were obtained from Shanghai cell bank of Chinese Academy of Science. The cells were cultured in Dulbecco’s modified Eagle medium (DMEM; Life Technologies, Grand Island, NY, USA) supplemented with 10% heat-inactivated fetal bovine serum (FBS; Gibco, Grand Island, NY, USA). Culture dishes were incubated at 37 °C in a humidified atmosphere containing 5% CO_2_, and subcultured approximately every 2~3 days. One day before transfection, HEK293T cells were transferred to 24-well plates. At 90% confluence, cells were transiently transfected using Lipofectamine3000 (Invitrogen, Carlsbad, CA, USA) at a ratio of 1 μL reagent with 1 μg total plasmid per well. Electrophysiological experiments were performed at 1~2 days after transfection.

### 4.2. Electrophysiological Recordings

Whole-cell voltage-clamp experiments were performed by using an Axon Multiclamp 700B Microelectrode Amplifier (Molecular Devices, Silicon Valley, CA, USA) at room temperature (21 °C–25 °C). Patch pipettes were fabricated from glass capillary tubes by PC-10 Puller (Narishige, Tokyo, Japan) with the resistance of 2~3 MΩ. Data acquisition and stimulation protocols were controlled by a Pentium III computer (Legend, Beijing, China) equipped with pCLAMP10.3 (Molecular Devices, Silicon Valley, CA, USA). Capacitance transients were cancelled. Cells with a seal resistance (Rseal) below 1 GΩ were omitted. Series resistance (Rs) was compensated (80%) to minimize voltage errors, and cells with an uncompensated series resistance (Rs) above 10 MΩ were omitted. Leak subtraction was performed using P/4 protocol. Data were lowpassed at 10 kHz. The rate of solution exchange was studied using solutions with different KCl concentrations and found to be about 95% complete within 20 s. The holding potential was −100 mV. Unless stated specially, all the recordings were done with the pulse of +40 mV.

### 4.3. Solutions and Drugs

In the patch-clamp recordings, the standard bath solution for hKv4.2 channels was consisted of the following (in mM): NaCl 125, KCl 2, MgCl2 1, glucose 10, HEPES 10, and TEA 20 (pH 7.4 titrated with NaOH). The standard bath solution for hKv1.3 channels was consisted of the following (in mM) NaCl 135, KCl 5, MgCl2 1, CaCl2 1.8, HEPES 10, and glucose 10 (pH 7.4 titrated with NaOH). Pipette solutions for both hKv4.2 and hKv1.3 were composed of the following (in mM): KCl 130, MgCl2 0.5, MgATP 2, EGTA 10, and HEPES 10 (pH 7.3 titrated with KOH).

BmP02 (purity ~98%, MW ~2950, Figure S2) and all of the mutants were purchased from TOP PEPTIDE, China, which was synthesized with a CEM microwave peptide synthesizer (Matthews, NC, USA) by the solid-phase method and cyclized by oxidative folding method. The synthetic BmP02 was dissolved in the bath solution, supplemented with 1 mg/mL bovine serum albumin (BSA) in order to prevent adherence of the toxin to the vials and the perfusion apparatus. Application of 1 mg/mL BSA alone did not alter Kv4.2, and Kv1.3 channel function. Unless otherwise stated, all reagents were purchased from Sigma. As for the bioactivity of synthetic BmP02, it was evaluated on Kv1.3 with EC50~32 nM, which indicates that synthetic BmP02 is a bioactive peptide similar to native BmP02 (EC50~7nM).

The sequence of BmP02 and all the mutants are as follows:

BmP02: VGCEECPMHCKGKNAKPTCDDGVCNCNV
BmP02 K11A: VGCEECPMHC**A**GKNAKPTCDDGVCNCNV
BmP02 K13A: VGCEECPMHCKG**A**NAKPTCDDGVCNCNV
BmP02EQ: VGC**QQ**CPMHCKGKNAKPTCDDGVCNCNV
BmP02DN: VGCEECPMHCKGKNAKPTC**NN**GVCNCNV.

### 4.4. 3D Modeling

Utilizing the X-ray crystal structures of KcsA Potassium channel (PDB: 1BL8) [32] as the templates, 3D model of Kv1.3 and Kv4.2 were generated using the Build homology models (MODELER) in Discovery Studio 4.0 (Accelrys, San Diego, CA, USA). The X-ray crystal structures of BmP02 (PDB: 1DU9) [15] and 3D model of Kv1.3 and Kv4.2 were used for docking analysis. Docking analysis was obtained by using ZDOCK in Discovery Studio 4.0 (Accelrys, San Diego, CA, USA).

### 4.5. Data Analysis

Data were analysed by Clamp Fit 10.3 (Molecular Devices, Silicon Valley, CA, USA) and Origin 8.5 (Originlab, Northampton, MA, USA). Results of data analysis were expressed as mean ± S.E.M, where and *n* represents the number of the cells examined. The Statistical significance was determined using the one-way ANOVA, and an asterisk denotes *p* < 0.05 unless otherwise stated.

Dose–response curve was drawn according to the Hill equation *I* = Im/(1 + ([*toxin*]/EC50)n), where Im is maximum of relative I20 (the current at 20 ms behind the peak current), and [toxin] is the concentration of BmP02. EC50 (half-maximal effective concentration) and *n* denote the toxin concentration of half maximal effect and the Hill coefficient, respectively.

Kv4.2 channel currents were elicited by the step pulses ranging from −100 to +60 mV for 200 ms with the increments of 10 mV (The holding potentials were held at −100 mV). For determining the voltage dependence of activation, the conductance was calculated using the formula: *G*(*V*) = *I*(*V*)/(V − ErK), where *I*(*V*) is the currents of Kv4.2 channel at the command voltage V, and ErK is the reversal potential. The conductance were normalized to the maximal value and the voltage dependence for activation of Kv4.2 channel fitted to a Boltzmann equation: *G*/*Gmax* = 1/(1 + exp(V − V^1/2^)/k), where V1/2 is the voltage for half-maximum activation and k is the slope factor. The inactivation kinetics was analysed by fitting the decay course of Kv4.2-mediated current to a single-exponential function: *I* = C + Aexp(−(*t* − t_0_)/τ), where t is time, t0 is the time when the currents were just starting to exponentially decrease, A represent the amplitudes of the channels inactivating with the time constant τ, and C is the steady-state asymptote approximating to the leakage.

The voltage-dependence of steady-state inactivation was determined by a 1000 ms conditional pulse step from −140 to +20 mV prior to a test pulse at +40 mV, and the data was described with the Boltzmann equation, *I*/*Imax* = 1/[1 + exp(*V* − V^1/2^)/k], where *V* is the membrane potential of the conditioning step, V^1/2^ is the membrane potential at which half-maximal inactivation is achieved, and k is the slope factor.

Recovery kinetics were analysed by a traditional two-pulse protocol consisting of a 1000 ms prepulse to +40 mV, followed by resting at −120 mV for the time in range of 0–600 ms with the increments of 30 ms, and a 200 ms test pulse to +40 mV. Recovery data were fitted with a single exponential equation: *I*/*I_peak_* = 1 − Aexp(−*t*/τ_rec_), where A is the relative proportion of current recovering with time constant τ_rec_.

Deactivation kinetics were analyzed by fitting the tail current after a 4 ms prepulse to +40 mV with a single-exponential function: *I* = C + Aexp(−(*t* − t_0_)/τ), where A represent the amplitudes of the channels deactivating with the time constant τ.

## Figures and Tables

**Table 1:** Parameters for voltage dependence of activation, inactivation with or without 1 μM BmP02. No significant difference between groups of control and BmP02 was observed, which was tested by One-way ANOVA. V_1/2_ is the voltage for half-maximal activation or inactivation. k is the voltage constant.

Group	Activation			Inactivation		
	V_1/2_ (mV)	k	*n*	V_1/2_ (mV)	k	*n*
Control	−13.87 ± 1.61	23.88 ± 1.90	10	−64.81 ± 1.73	6.86 ± 1.29	10
BmP02	−14.23 ± 1.76	23.07 ± 2.05	10	−62.99 ± 1.81	5.93 ± 1.30	10

**Table 2:** Parameters for recovery in presence or in absence of BmP02. * *p* < 0.05; *** *p* < 0.001; significant difference between control and BmP02 was tested by One-way ANOVA; τ is the time constant of recovery.

Constants	Control	Concentration of BmP02		
		100 nM	1 μM	3 μM
τ (ms)	189.35 ± 10.87	177.15 ± 15.41	109.58 ± 18.10 *	79.45 ± 5.62 ***
*n*	10	10	10	10

## Supplementary Materials

The following are available online at www.mdpi.com/2072-6651/8/10/280/s1. Figure S1: Homology modeling of Kv4.2 and Kv1.3 using the structure of KcsA (PDB: 1BL8) as template; Figure S2: The purity and the molecular weights of synthetic BmP02; Table S1: The primers used in the construction of mutants.
