# Supplementary material for: BmP02 Atypically Delays Kv4.2 Inactivation: Implication for a Unique Interaction between Scorpion Toxin and Potassium Channel
Source: Toxins (Basel). 2016 Sep 27;8(10):280. doi: 10.3390/toxins8100280 (PMC5086640; doi:10.3390/toxins8100280)
Supplement: Supplementary file 1 [file toxins-08-00280-s001.pdf]

Supplementary Materials: BmP02 Atypically Delays Kv4.2 Inactivation: Implication for a Unique Interaction Between Scorpion Toxin and Potassium Channel

Bin Wu, Yan Zhu, Jian Shi, Jie Tao and Yonghua Ji

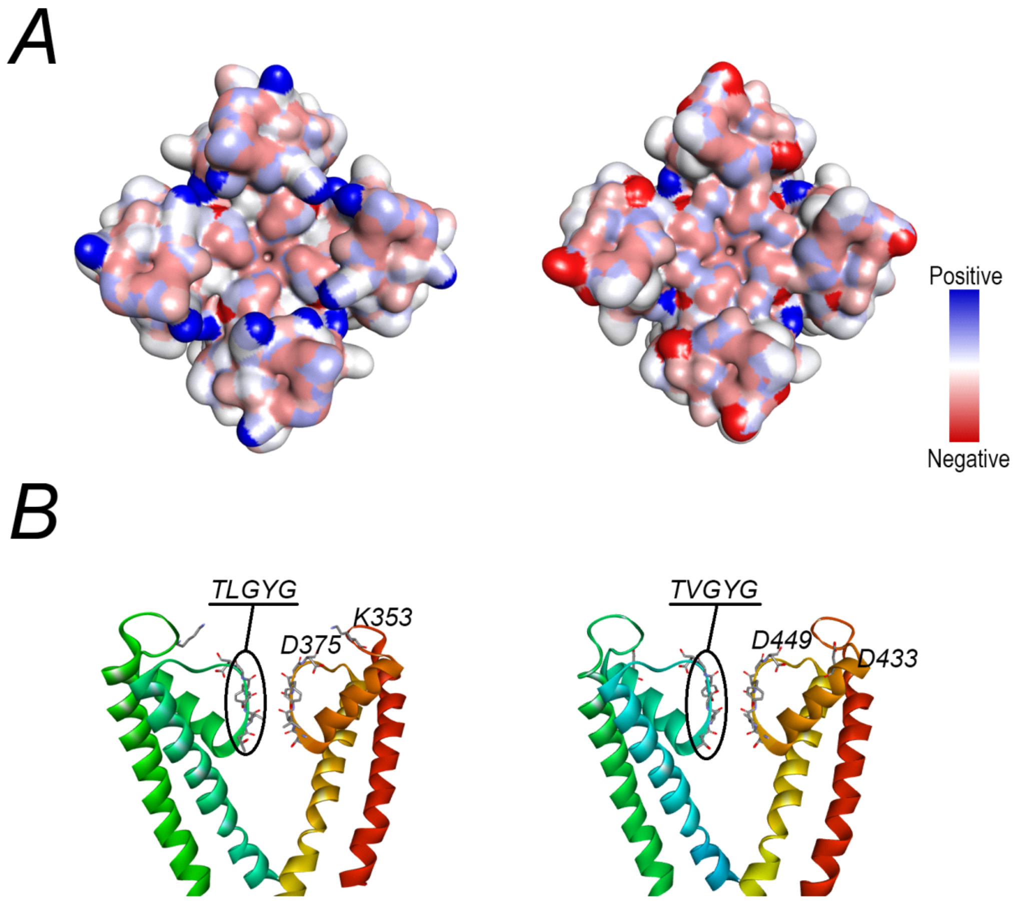

Figure S1. Homology modeling of Kv4.2 and Kv1.3 using the structure of KcsA (PDB: 1BL8) as template. (**A**) A vertical view of Kv4.2 (left) and Kv1.3 (right). The positively charged groups are shown in blue and the negatively charged groups are in red; (**B**) An insight into the structure of Kv4.2 (left) and Kv1.3 (right). Residues in the selectivity filter and a Asp nearby are highlighted. K_{353} in Kv4.2 and D433 (the corresponding residue of A_{359} in Kv4.2) in Kv1.3 are also highlighted.

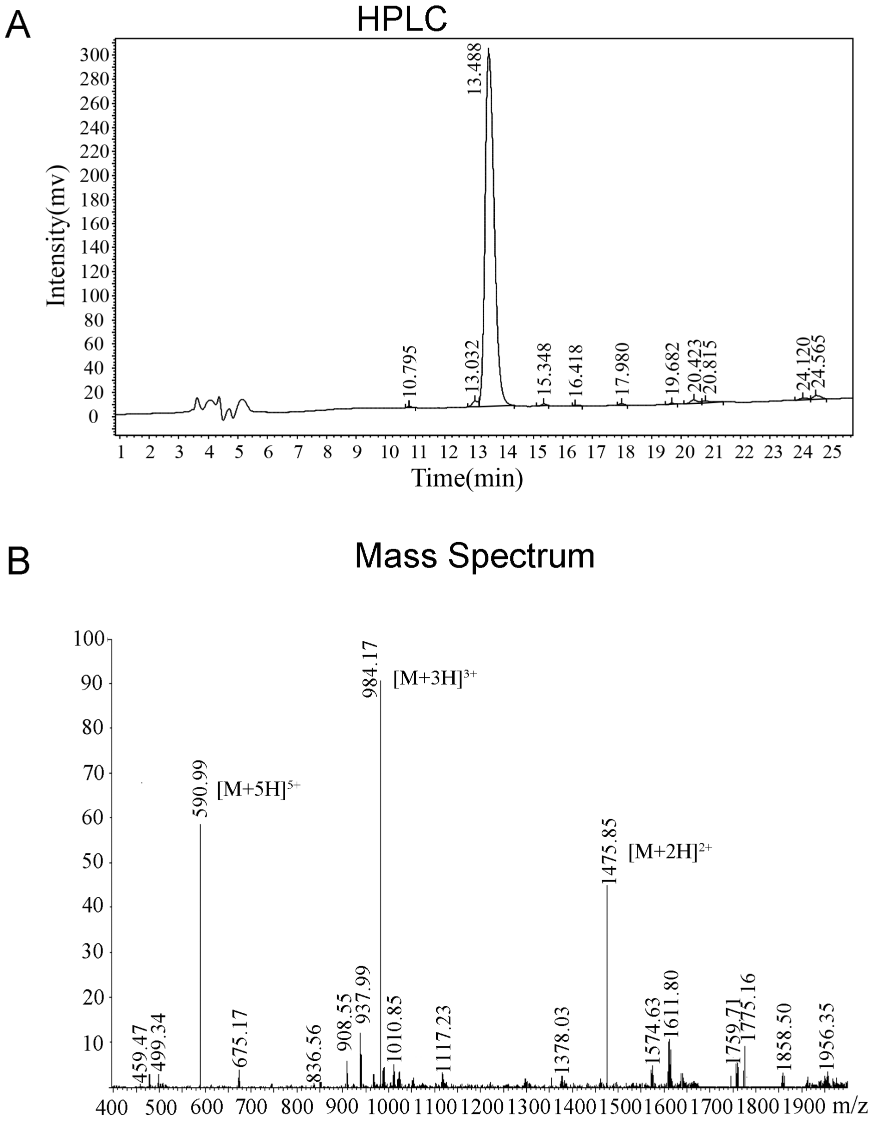

Figure S2. The purity and the molecular weights of synthetic BmP02. (**A**) Purity of synthetic BmP02 was determined by High Performance Liquid Chromatography (HPLC); (**B**) Molecular weights of the peptides were determined by mass spectrum (MS).

Table S1. The primers used in the construction of mutants. The mutated sites are underlined. S: sense, A: anti-sense.

Name	Mutated Sides	Primer
Kv4.2M1	$A_{359}D$	S:5′-CAGCATCCCTGACGCCTTCTGGTATACCATCGT<br>A:5′-GGTATACCAGAAGGCGTCAGGGATGCTGGTGAA
Kv4.2M2	$K_{347}A/K_{353}G$	S:5′-GCGGGGTCTTCGGCTAGCGGGTTCACCAGCATCCCT<br>A:5′-CCCGCTAGCCGAAGACCCCGCCTCTGCGTAGAACAT
Kv4.2M3	$K_{347}A/K_{353}G/A_{359}D$	S:5′-GCGGGGTCTTCGGCTAGCGGGTTCACCAGCATCCCTGACGCCTTCTGGTA<br>TACCATCGT<br>A:5′-GTCAGGGATGCTGGTGAACCCGCTAGCCGAAGACCCCGCCTCTGCGTAG<br>AACATAACTG
